# A Bioinformatics Study of Ropivacaine plus Dexamethasone Prolonging the Duration of Nerve Block

**DOI:** 10.1155/2022/5869103

**Published:** 2022-08-11

**Authors:** Yongjie Chen, Jin Wu, Jingjing Xu, Yijun Chen, Changshun Huang

**Affiliations:** Department of Anesthesiology, Ningbo First Hospital, No. 59 Liuting Street, Ningbo 315010, Zhejiang, China

## Abstract

The study focuses on the potential function of dexamethasone on ropivacaine in sciatic nerve blocks. Nine Sprague–Dawley (SD) rats were randomly divided into three groups: normal group (NG), control group (CG), and experimental group (EG), with three rats in each group. The CG was injected with diluted ropivacaine (0.5% concentration); the EG was injected with a diluted ropivacaine+dexamethasone mixture, and the NG was injected with an equal amount of saline. The sciatic nerve in the thigh was collected for sequencing two days after injection in each group. Differential analysis was performed for NG-vs-CG, NG-vs-EG, and CG-vs-EG based on the sequencing dataset. The modular genes associated with ropivacaine and ropivacaine+ dexamethasone were screened by weighted coexpression network analysis (WGCNA), differentially expressed modules among them were enriched for analysis, and protein-protein interaction (PPI) networks were constructed to observe high and low expression among key genes in immune cells. Twenty-two and three differential genes associated with ropivacaine (green-yellow module) and ropivacaine+dexamethasone (palevioletred3 module) were acquired, respectively, which played important roles in biological processes such as erythrocyte homeostasis, erythroid differentiation, and hemoglobin metabolic processes. PPI revealed that AHSP, ALAS2, EPB42, HBB, and SLC4A1 were interacting and the expression of these five genes was upregulated in the CG compared with the NG, while the expression of them was downregulated in the EG compared with the CG. The immunological analysis also showed significant differences in the expression of various immune cells in the 3 groups. AHSP, ALAS2, EPB42, HBB, and SLC4A1 are genes associated with hemoglobin, and dexamethasone combined with ropivacaine may prolong anesthesia by affecting local vasoconstriction to some extent.

## 1. Introduction

Peripheral nerve blocks are frequently applied in various surgical procedures for surgical anesthesia and postoperative pain. Although local anesthetic drugs are clinically available for analgesia, their comparatively short half-life and possible acute toxicity to the cardiovascular system and central nervous system limit the dose for postoperative pain management [1]. Reportedly, one strategy of continuous infusion of ropivacaine at low concentrations can reduce postoperative pain [2]. Ropivacaine is a unique highly potent amide local anesthetic with a levorotatory structure that has low lipid solubility and cardiac and central nervous system toxicity [3]. It presents some similar mechanism of action to other local anesthetics, as it suppresses sodium influx in nerve fibers in a reversible way. The amide preferentially binds and inactivates sodium channels in the open status, thus preventing the propagation of action potentials [4].

A single injection of ropivacaine can only deliver a brief analgesic effect that cannot meet clinical needs. To date, several methods have been applied to prolong the duration of analgesia, including intermittent injections, continuous administration via catheter, increasing the concentration or dose administered, and supplementation with epinephrine, dexamethasone, or dexmedetomidine [5]. Dexamethasone is one potent, long-lasting glucocorticoid with analgesic, antiemetic, and anti-inflammatory properties, which may act as one adjuvant to local anesthetics in chronic pain management by suppressing the release of inflammatory mediators, lessening ectopic neuronal firing, and suppressing firing of nociceptive C-fibers medicated through potassium channel [6]. Just one study showed that ropivacaine combined with dexamethasone for supraclavicular brachial plexus nerve block prolonged motor and sensory block [7]. It has also been shown that in the use of craniotomy, scalp infiltration with ropivacaine combined with dexamethasone delivered better postoperative analgesia than ropivacaine alone [8]. However, less is known about the mechanism of action of ropivacaine alone or its combination with dexamethasone. The present study, however, used bioinformatic analysis to investigate the potential mechanisms of ropivacaine or ropivacaine combined with dexamethasone in the sciatic nerve.

## 2. Methods

### 2.1. Drug Treatment

Nine Sprague–Dawley (SD) rats were randomly assigned to three groups: normal group (NG), control group (CG), and experimental group (EG). The drugs for the groups were formulated as follows: NG was injected with saline. Drugs for CG were prepared as 6.7 ml of ropivacaine stock solution + 3.3 ml of saline. Drugs for EG were prepared as 6.7 ml ropivacaine stock solution + 3.3 ml saline + 500 ul dexamethasone injection. Three rats in every group were anesthetized through 2% phenobarbital sodium (3 ml/kg), followed by intrathecal administration of 0.1 ml drug into the sciatic nerve. The sciatic nerve in the thigh of SD rats was collected for sequencing (Azenta Ascent) two days after injection in each group of rats.

### 2.2. Analysis of Differences

The data sets of NG-vs-CG, NG-vs.-EG, and CG-vs.-EG were analyzed using the “limma” package in *R*. Differentially expressed genes (DEGs) were those meeting the screening criteria of |log2 (FC)|>1 and *p* · adj < 0.05. Volcanoes and circular heat maps were plotted via the *R* package “ggplot2.”

### 2.3. Weighted Coexpression Network (WGCNA) Analysis

Coexpression networks were constructed using the *R* software and the WGCNA package. The pickSoft threshold function was used to calculate soft thresholds through scale independence of modules and average connectivity analysis. Then, hierarchical clustering was performed to identify coexpression modules to obtain a hierarchical clustering tree. Finally, the minimum quantity of module genes was set to 30, followed by the merging of the second module based on correlation.

### 2.4. Enrichment Analysis

Through Gene Ontology (GO) annotation and Kyoto Encyclopedia of Whole Genes and Genomes (KEGG) enrichment analysis with the DAVID database (https://david.ncifcrf.gov/), the potential functions of genes were predicted. Bubble maps were drawn using the *R* “ggplot2” software package based on *p* values. Network visualization was conducted via the *R* “cluster profile” package. *p* < 0.05 indicates a statistically significance.

### 2.5. Establishment of Protein-Protein Interaction (PPI) Network

PPI networks of identified genes were obtained using STRING (https://string-db.org/), a search tool for retrieving interacting genes/proteins.

### 2.6. Immunoinfiltration Analysis

The immune infiltration in each sample was analyzed using the online tool ImmuCell AI (https://bioinfo.life.hust.edu.cn/ImmuCellAI#!/analysis) for analysis. Through the *R* package “ggplot2,” the proportion of immune cells in each group was mapped. Expression analysis and plotting were performed via Graphpad Prism 8, and *p* < 0.05 was deemed significant in a statistical sense.

## 3. Results

### 3.1. Differential Analysis

**Figure 1:**
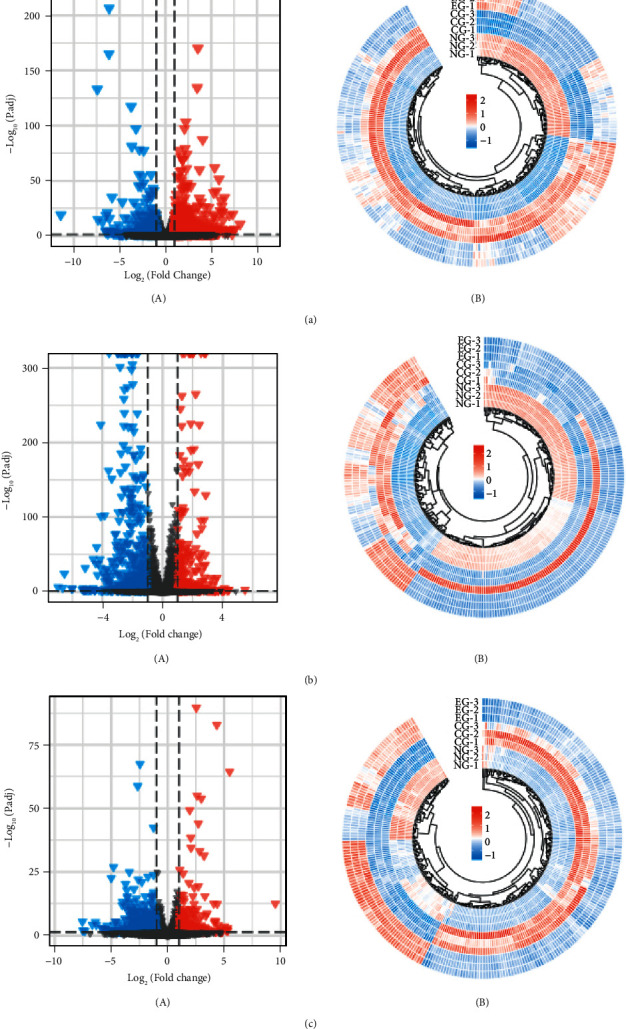
Screening for differentially expressed genes (DEGs) in and/or combined dexamethasone in the sciatic nerve. (a) DEGs between the two groups of the normal group (NG)-vs.-control group (CG, A) and their expression in each sample (B). (b) DEGs between the two groups of NG-vs.-experimental group (EG, A) and their expression in each sample (B). (c) DEGs between the two groups of CG-vs.-EG (A) and their expression in each sample (B). (d) DEGs between the two groups of NG-vs.-CG (A) and their expression in each sample (B). (e) DEGs between the two groups of NG-vs.-EG (A) and their expression in each sample (B).

First, differential expression analysis was conducted for NG-vs-CG, NG-vs-EG, and CG-vs-EG, respectively. The filtering condition was |log2(FC)|>1, *p* · adj < 0.05. The results showed that 1203 DEGs were screened between the two groups of NG-vs-CG, including 867 upregulated genes and 336 downregulated ones. Totally, 830 DEGs were screened between the two groups of NG-vs-EG, including 337 upregulated genes and 503 downregulated ones, and 732 DEGs were screened between the two groups of CG-vs-EG, including 259 upregulated genes and 473 downregulated genes. In addition, heat maps were drawn to present the expression of the top 300 genes in individual samples (see Figure 1).

### 3.2. WGCNA Analysis

**Figure 2:**
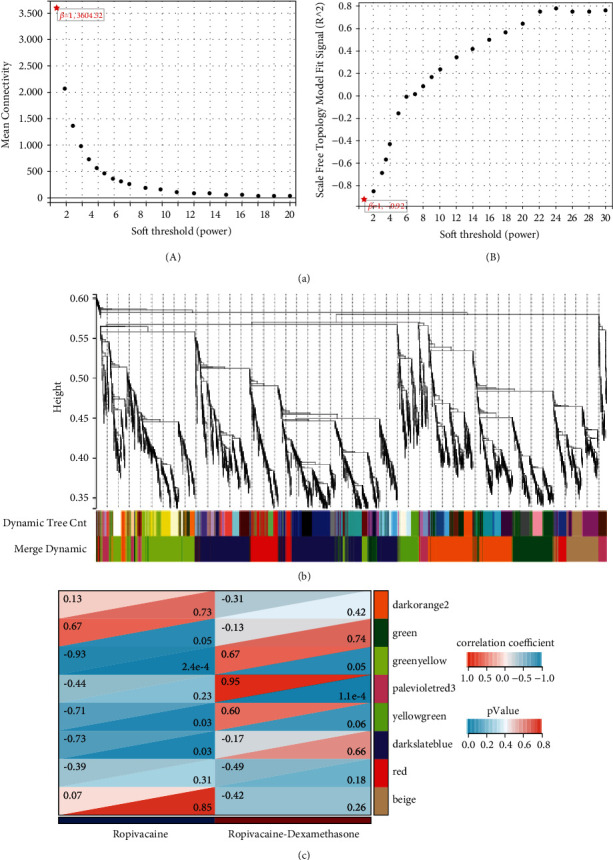
Weighted coexpression network analysis (WGCNA) analysis. (a) Mean connectivity (A) and standard independence (B). (b) Gene clustering plot. (c) Heat map of correlation between modules and traits.

A WGCNA analysis was performed on the whole genes to screen the modular genes with the strongest correlation with ropivacaine and ropivacaine+dexamethasone. The analysis showed that genes enriched in the green-yellow module were significantly negatively correlated with ropivacaine (*p* < 0.05, *r* = −0.93); genes enriched in the palevioletred3 module were significantly positively correlated with ropivacaine + dexamethasone (*p* < 0.05, *r* = 0.95) (see Figure 2).

### 3.3. Intersecting Genes

**Figure 3:**
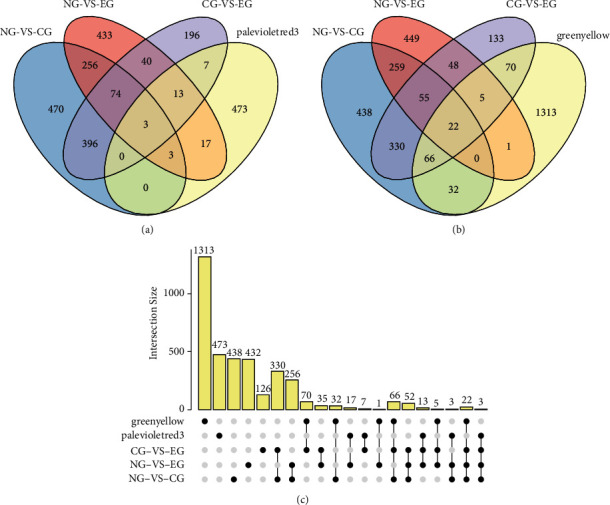
Intersecting genes. (a) Intersection of three groups of DEGs with palevioletred3 module genes. (b) Intersection of three groups of DEGs with green-yellow module genes. (c) Upset plot showing the intersection between groups.

Then, NG-vs.-CG, NG-vs.-EG, and CG-vs.-EG DEGs were let to intersect with green-yellow module and palevioletred 3 module genes, respectively. The results showed that there were three overlapping genes between the three DEGs and the palevioletred3 module, and 22 overlapping genes between the three DEGs and the green-yellow module. Upset plots were used to show the intersection between the groups more clearly (see Figure 3).

### 3.4. Enrichment Analysis

**Figure 4:**
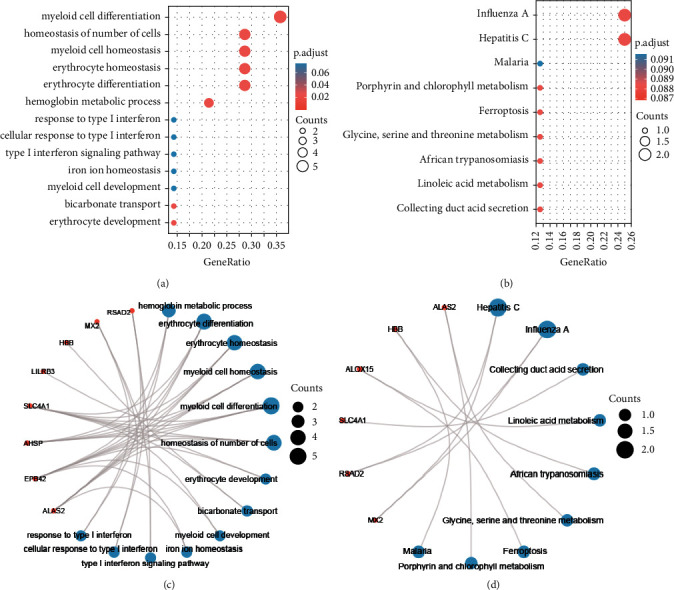
Gene Ontology (GO) and Kyoto Encyclopedia of whole Genes and Genomes (KEGG) analysis of overlapping genes. (a) Bubble diagram of GO analysis. (b) Bubble diagram of KEGG analysis. (c) Visualization of GO analysis network. (d) Visualization of KEGG analysis network.

In addition, GO and KEGG analyses were conducted on these 22 + 3 overlapping genes to explore the biological processes in which they were involved. The results of GO analysis showed that they played crucial roles in biological processes such as myeloid differentiation, cell number homeostasis, myeloid homeostasis, erythroid homeostasis, erythroid differentiation, and hemoglobin metabolic processes. The KEGG analysis results revealed their involvement in the secretion of collecting ductal acid. The KEGG analysis results also showed their involvement in the pathways of collector acid secretion, linoleic acid metabolism, and metabolism of glycine and serine, as well as threonine (see Figure 4).

### 3.5. PPI and Expression Analysis

**Figure 5:**
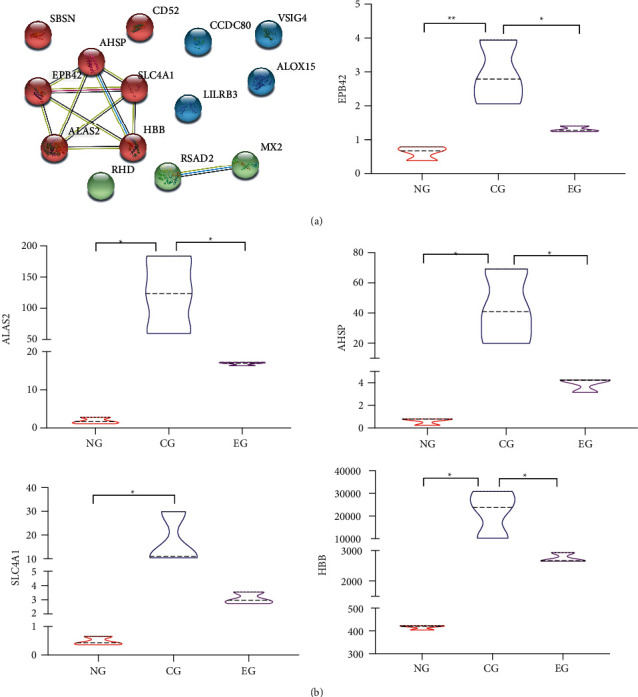
Protein-protein interaction (PPI) and expression analysis. (a) PPI network diagram of overlapping genes. (b) Expression of genes with reciprocal relationships in every group. ^*∗*^*p* < 0.05; ^*∗∗*^*p* < 0.01.

Then PPI networks were constructed for these 22 + 3 overlapping genes, and the results showed that AHSP, ALAS2, EPB42, HBB, and SLC4A1 had reciprocal relationships. Then, their expressions in the NG, CG, and EG were evaluated, respectively. As a result, the expression of these 5 genes was upregulated in the CG compared with the NG, while the expression of them was downregulated in the EG compared with the CG (see Figure 5).

### 3.6. Immunoinfiltration Analysis

**Figure 6:**
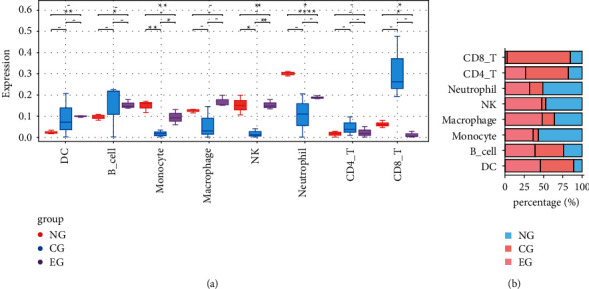
Immune infiltration analysis of each group. (a) Level of immune cell infiltration in each group. (b) Superimposed scale plot showing the percentage of different immune cells in each group.

Immunological observation is important in the analysis of tissue or nerve injury. Therefore, immune cell infiltration in the three groups was observed. The superimposed proportion plot clearly showed that compared with the NG, the percentage of CD4+ T cells and CD8+ T cells elevated in the CG and then decreased in the EG; the proportion of neutrophils, NK cells, macrophages, and monocytes decreased in the CG and then increased in the EG (see Figure 6).

### 3.7. Differences in Immune Cells in High and Low Gene Expression Groups

**Figure 7:**
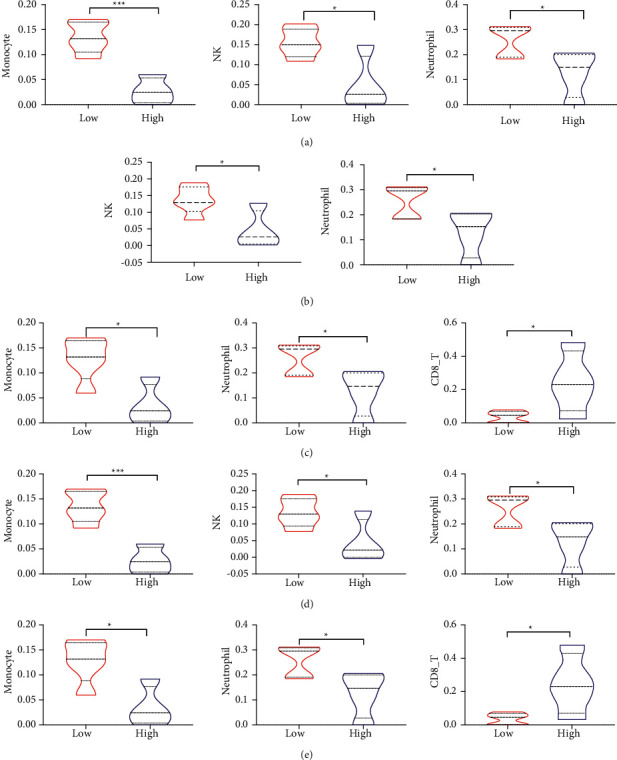
Differences between the high and low gene expression groups in terms of immune cells. (a) Immune cells with differences in AHSP high- and low-expression groups. (b) Immune cells with differences in ALAS2 high- and low-expression groups. (c) Immune cells with differences in EPB42 high- and low-expression groups. (d) Immune cells with differences in HBB high- and low-expression groups. (e) Immune cells with differences in SLC4A1 high- and low-expression groups.

Based on the median value of gene expression, the genes were assigned to high- or low-expression groups and the differences in immune cells between the two groups were explored. According to the results, compared with the low-expression group, monocytes, NK cells, and neutrophils were less expressed in the AHSP and HBB high-expression group; NK cells and neutrophils were less expressed in the ALAS2 high-expression group; monocytes and neutrophils were less expressed in the EPB42 and SLC4A1 high-expression group, and CD8+ T cells were highly expressed in the EPB42 and SLC4A1 high-expression group (see Figure 7).

## 4. Discussion

Dexamethasone, as an adjuvant, provides better nerve blockade when combined with local anesthetics alone. One researcher evaluated over 1,000 peripheral nerve blocks and discovered a median block duration of 15 h with ropivacaine alone in contrast to 22 hours when 4 /8 mg perineural dexamethasone was additionally used [9]. In addition, they lowered the intensity of pain at what surgical patients mentioned as the worst pain point after the block and improved patient satisfaction with postoperative pain management [10]. It has been suggested that this may be due to the ability of glucocorticoids to cause local vasoconstriction in individuals to slow the absorption of local anesthetic drugs. On the other hand, dexamethasone has a complex spatial structure, and their combination could prolong the release process of this drug, thus providing better analgesia and sedation [11]. However, the exact mechanism is still unknown to us. To explore the potential mechanism of their action, differential expression analysis was performed for NG-vs.-CG, NG-vs.-EG, and CG-vs.-EG, respectively, and then based on the results of this analysis, 22 + 3 overlapping genes were acquired by intersections with the ropivacaine-associated green-yellow module gene and the ropivacaine + dexamethasone-associated palevioletred3 module gene. Enrichment analysis also showed that these genes played important roles in maintaining cellular homeostasis. Then, PPI networks were constructed for these 22 + 3 overlapping genes, and the results showed that AHSP, ALAS2, EPB42, HBB, and SLC4A1 had reciprocal relationships.

Erythrocytes contain large amounts of AHSP, which specifically accompanies and stabilizes *α*-Hb hemipigments. AHSP promotes histidinyl coordination of heme iron to stabilize the ferrous and trivalent forms of free *α*-Hb, thereby inhibiting the production of harmful reactive oxygen species by *α*-Hb and preventing oxidative stress-induced precipitation [12]. In addition, AHSP plays an important role in the physiological processes that regulate vascular carbon monoxide concentration [13]. ALAS2 is the first rate-limiting enzyme in the erythrocyte heme biosynthesis pathway [14] and may function as a modifier gene [15]. Its overexpression causes transgenic mice to exhibit muscle atrophy, which is associated with muscle mitochondrial dysfunction induced by ALA accumulation [16]. HBB is one member of the bead protein family, a group of structurally conserved proteins that usually contain a heme group and are capable of reversibly binding oxygen and other gaseous ligands in erythrocytes [17]. In addition to its oxygen transport function, it exhibits antitumor responsiveness and can modulate tumor progression [18]. However, it is also found in many nonerythroid cells, such as activated macrophages, alveolar type II epithelial cells, and thylakoid cells [19]. SLC4A1 is an amino acid glycoprotein responsible for the rapid electroneutral exchange of chloride ions and bicarbonate across the plasma membrane, a process that increases the ability of blood to carry carbon dioxide in the form of plasma bicarbonate [20]. In the present research, the expression analysis revealed that the expression of these 5 genes was upregulated when ropivacaine was used alone and it was knocked down when dexamethasone was coadministered. Although no clear studies verify a direct effect of ropivacaine and dexamethasone on them, it can be found that these genes have a crucial part during hemoglobin metabolism. The regulation of hemoglobin has an important role in vasodilation and contraction. Some studies have shown that local anesthetics also have a direct effect on blood vessels. Except for cocaine, these drugs are commonly thought to cause vasodilatory effects. In contrast, local anesthetics such as dexamethasone coadministered with ropivacaine induce local vasoconstriction, thereby lowering the rate of drug absorption and extending the duration of obstruction [21]. Whereas the exact mechanism of the effects involved remains to be studied in more depth.

One study found that dexamethasone, one adjuvant to local anesthetics, prevented local anesthesia-induced reversible neurotoxicity and short-term “rebound nociceptive hypersensitivity” in one mouse model of a sciatic nerve block [22]. Dexamethasone combined with local anesthesia is indicated to have good side effects. It is known that tissue injury is nearly inevitable in surgery, which activates a systemic inflammatory reaction and gives rise to changes in the endocrine and metabolic systems. Anesthesia also has an important impact on the immune status [23]. Glucocorticoids are highly effective and commonly used as anti-inflammatory agents that can act on macrophages by a complex mechanism of direct and indirect transinhibition or transactivation of genes mediated by the GC receptor (GR). Moreover, the GC-induced phenotype can exhibit not only a reduction in inflammatory activity but also an induction of processes related to inflammation regression and wound healing [24]. It has been shown that dexamethasone increases the KLF9 level via GR and increases the production of mitochondrial reactive oxygen species, leading to mitochondria-dependent apoptosis of macrophages. Also, it was proposed that reducing KLF9 levels could attenuate dexamethasone-induced macrophage apoptosis [25]. Immunoassay in the present study showed that ropivacaine application increased the percentage of CD4+ T cells, CD8+ T cells, and lowered the proportion of neutrophils, NK cells, monocytes, and macrophages; and the addition of dexamethasone reversed them. In addition, monocytes, macrophages, and NK cells were differentially expressed between high- and low-expression groups of genes. It has been demonstrated that the synthesis of proteins related to hemoglobin is present during monocyte-macrophage differentiation and decreases as differentiation proceeds [26]. It is suggested that ropivacaine and dexamethasone may modulate immune cells by regulating AHSP, ALAS2, EPB42, HBB, and SLC4A1 and thus reduce the incidence of adverse effects. However, this is only our speculation, and the exact mechanism remains to be investigated.

## 5. Conclusion

In conclusion, ropivacaine combined with dexamethasone may cause certain local vasoconstriction by affecting AHSP, ALAS2, EPB42, HBB, and SLC4A1, thus slowing the absorption of local anesthetics and prolonging the duration of nerve block, but the present study is only based on data for correlation analysis, so more in-depth studies are needed for verification.

## Data Availability

The data used to support the findings of this study are available from the corresponding author upon request.
